# Critical Considerations on Polyurethane-Coated Implants in Breast Reconstruction

**DOI:** 10.1093/asj/sjaf171

**Published:** 2025-08-23

**Authors:** Manuel Cabrera Charleston, Daniela Guadalupe Oscura Paredes

We read with great interest the article by Catanuto et al, titled “One-Stage Implant-Based Breast Reconstruction With Polyurethane-Coated Device: Standardized Assessment of Outcomes.”^1^ We commend the authors for their structured approach in evaluating the outcomes of direct-to-implant (DTI) breast reconstruction with polyurethane-coated implants, using internationally recognized criteria, such as the Clavien–Dindo classification and Association of Breast Surgery-British Association of Plastic, Reconstructive, and Aesthetic Surgery (ABS-BAPRAS) indicators.^2^

This study adds meaningful data to the literature on prepectoral, acellular dermal matrix (ADM)-free breast reconstruction. The reported absence of capsular contracture (Baker grade 3-4) and implant malposition is noteworthy, especially considering the patient cohort included individuals with previous radiotherapy, moderate ptosis, and neoadjuvant chemotherapy.

## COMPLICATION RATES AND QUALITY ASSURANCE TARGETS

The implant loss and unplanned reoperation rates slightly exceeded ABS-BAPRAS benchmarks.^3^ Although the authors suggest a learning curve and the use of the italic-S incision as contributing factors, a more detailed stratification of complications by incision type, BMI, and patient risk profile (eg, smoking and diabetes) would be valuable. Such analysis could help refine selection criteria for polyurethane-based DTI approaches.

## PATIENT-REPORTED OUTCOMES

Although the study acknowledges the absence of routine patient-reported outcomes, the inclusion of tools, such as the BREAST-Q, would strengthen the assessment of satisfaction, body image, and quality of life.^4^ Given that nearly one-third of patients sought revision due to cosmetic concerns, subjective outcomes would complement the objective clinical metrics.

## POLYURETHANE SAFETY AND BREAST IMPLANT-ASSOCIATED ANAPLASTIC LARGE CELL LYMPHOMA CONSIDERATIONS

The discussion surrounding breast implant-associated anaplastic large cell lymphoma (BIA-ALCL) and surface texturing is important.^5^ Despite the low incidence associated with Polytech implants, the study might benefit from outlining patient follow-up protocols or planned surveillance strategies, considering the long latency period of BIA-ALCL.

## NEED FOR COMPARATIVE EVIDENCE

The authors reference a study comparing ADM to polyurethane implants, yet the lack of statistically significant results and randomization limits definitive conclusions. We believe prospective comparative trials—ideally multicentric—are crucial to establishing evidence-based algorithms for reconstructive planning.^6^

In conclusion, we agree that polyurethane-coated implants represent a promising tool in 1-stage breast reconstruction. Their role in improving pocket stability and reducing capsular contracture is compelling. Nevertheless, long-term data and the integration of patient-centered outcomes are necessary to validate this approach as a standard of care.
